# Aging, Alzheimer’s Disease and Dysfunctional Glycolysis; Similar Effects of Too Much and Too Little

**DOI:** 10.14336/AD.2019.0611

**Published:** 2019-12-01

**Authors:** Alan R Hipkiss

**Affiliations:** Aston Research Centre for Healthy Ageing (ARCHA), School of Life and Health Sciences, Aston University, Birmingham, U.K.

**Keywords:** glycolysis, triosephosphate isomerase, methylglyoxal, glycation, erythrocytes, alpha-synuclein

## Abstract

Aging and much related dysfunction can be delayed by decreased glycolysis, however dysfunctional glycolysis appears to play a causative role in Alzheimer’s disease (AD). It is proposed here that this apparent contradiction can be reconciled by suggesting that both over-use and inhibition of the glycolytic enzyme triosephosphate isomerase can limit NADH generation and increase protein glycation. It is also suggested that excessive glycolysis in erythrocytes may provide a source of systemic methylglyoxal and glycated alpha-synuclein, both of which accelerate aging onset and neurodegeneration.

Two recent papers have highlighted the likelihood that glycolytic dysfunction plays an important causative role in onset and/or progression of Alzheimer’s disease (AD) [1,2]. The suggestion that glycolysis may be an important determining factor with respect to age-related disorder is not new [3,4], as much research has shown that excessive glycolytic activity seems to accelerate aging onset, whereas decreased glycolysis delays aging [4]. The suggestion that glycolytic dysfunction provokes age-related neurodegeneration, while partial suppression of glycolysis delays much age-related change, appears somewhat contradictory. It is suggested that these observations can be reconciled as follows.

## Role of triose-phosphate isomerase

The explanation centres on the glycolytic enzyme, triose-phosphate isomerase (TPI), mutations in which have been shown to be associated with neurological dysfunction and synaptic vesical impairment [5]. 30 years ago, it was shown by Gracy and co-workers [6,7] that TPI was not a true catalyst, because its structure alters as a result of its catalytic action. TPI normally catalyses the conversion of dihydroxyacetone phosphate (DHAP) to glyceraldehyde-3-phosphate (G3P). It was found that as a consequence of its catalysis, two asparagine residues (15 and 71) increase their tendency to spontaneously deamidate into aspartic acid residues, the consequence of which is decreased TPI activity due to disassociation of the dimeric enzyme and the proteolysis of the resultant monomers [8]. Indeed, it has been stated that “the probability of deamidation of an individual TPI molecule is a function of the number of times it is used as a catalyst” [9]. Whilst insufficient TPI activity does not totally prevent glycolytic flux, a decrease in G3P formation is one consequence, which would decrease the amount of NAD reduced to NADH mediated in the succeeding step, catalysed by glyceraldehyde-3-phopshate dehydrogenase). The Theurey et al. paper [2] suggests that insufficient NADH generation is responsible for the mitochondrial hypo-metabolism in AD, and this (i.e. decreased glyceraldehyde-3-phosphate generation) would be one outcome of TPI insufficiency. Importantly, however, there is another potentially deleterious outcome because accumulation of DHAP would also occur. Not only is DHAP an effective glycating agent, but it can spontaneously decompose into methylglyoxal (MG) which is even more reactive. Indeed, MG is thought to be responsible for much age-related formation of advanced glycation end-products (AGEs) which characterise aged cells and tissues, including the brains of AD patients [10,11].

### Neurological dysfunction, methylglyoxal and triosephosphate isomerase

Furthermore, there are additional sources of TPI dysfunction. TPI can be inactivated by nitration induced by the beta-amyloid peptide (βA4) [12], which accumulates in the AD brain; again, consistent with the proposal that lowered TPI activity (in either or both neurons and astrocytes) may be contributory to AD pathology [13]. Additionally, the tau protein normally protects TPI against oxidative damage, but upon forming paired helical filaments following tau phosphorylation (as is found in the AD brain), this protective activity is lost [14]. Thus, it may be concluded that the aberrant protein forms which characterise the AD brain can also contribute to TPI dysfunction and its ongoing consequences. An additional source of TPI dysfunction has recently been revealed which is that certain commonly used phenylpyrrole fungicides appear to inhibit TPI activity and also provoke MG accumulation [15]. Although it remains to be confirmed whether these fungicides enter the human body, including the brain and erythrocytes, their action on TPI must be of concern with respect to human neurodegenerative conditions.

### Potential deleterious consequences of excessive erythrocyte glycolysis.

In contrast to most cells in the human body, erythrocytes are uniquely glycolytic as they do not possess mitochondria and cannot synthesise new proteins. It is therefore suggested that, as a consequence of the activity-induced decreased TPI activity, excessive and persistent erythrocytic glycolysis is likely to contribute to age-related disorders generally. It is interesting to note that TPI activity in human erythrocytes is normally at least 3-fold greater than any other glycolytic activity [*16*]. This possibly reflects an evolutionary adaptation to compensate for the decline in TPI activity which may normally occur during the erythrocyte lifespan and thereby prevents TPI activity becoming rate-limiting. However, the human (Western) diet has altered considerably during the very recent past; the current Western diet contains a huge increase in carbohydrate content compared the “hunter-gather” diet to which humankind has presumably evolutionarily adapted. Thus, it is likely that erythrocytes of humankind are ill-equipped to cope with current Western diet which can induce a high and almost persistent glycolytic flux. Such a condition is likely to provoke MG accumulation in erythrocytes, due to diet-induced TPI deficiency as described above. Although erythrocytes also contain glyoxalase activity which should remove excess MG, this activity has also been shown to decline with erythrocyte age [*17*]. Not only would excessive red cell glycolysis and MG accumulation provoke glycation of erythrocytic proteins, but systemic MG distribution might occur as MG can also provoke red cell lysis (eryptosis) [*18*]. This proposal may explain why high glycaemic index diets are generally deleterious causing, for example, not only extensive MG-induced macromolecular modification but also proteostatic dysfunction [19], thereby compromising the selective elimination of aberrant protein forms, another characteristic of the aged phenotype.

It is suggested that excessive glycolysis in older erythrocytes, where glyoxalase activity has declined, may provoke a situation in which not only does MG accumulate but systemic MG distribution may occur. In addition, intra-erythrocyte MG-induced glycation of alpha-synuclein is also possible, thus raising the possibility that glycated alpha-synuclein might also be systemically distribusted by similar older and highly glycolytic erythrocytes. This may have implications for Parkinson’s disease, a major characteristic of which is the accumulation of the alpha-synuclein protein in the form of Lewy bodies, especially when the protein is glycated [*20*]. Alpha-synuclein is also found in erythrocytes and is readily susceptible to glycation [20]. Thus, it is likely that excessive glycolysis within older red cells, where glyoxalase activity has declined [*17*], increased amounts of MG may be generated to provide not only a systemic source of MG but also glycated alpha-synuclein. Given that it has been suggested that alpha-synuclein may possess prion-like properties [*22*], one can begin to understand the relationship between type-2 diabetes and neurological disorders including Parkinson’s disease. Such a relationship is also indicated by the detection of the neurotoxin 1-acetyl-6,7-dihydroxyl-1,2,3,4-tetra-hydroisoquinoline (ADTIQ) in the brains of type-2 diabetics and Parkinson’s disease patients: ADTIQ is formed by the spontaneous reaction of MG with dopamine [23].

## Conclusions

Glycolysis is not benign. Many studies have shown that excessive glycolysis is indeed deleterious to health and can accelerate the onset of age-related dysfunction and aging generally [24, 25, 26]. It proposed that, the energy requirements of the AD brain might be compromised by the decrease in NADH synthesis consequent upon a decline in TPI catalytic activity caused by excessive glycolytic activity in the brain, plus the effects of accumulation of certain aberrant protein molecules (beta-amyloid peptide-induced nitration of TPI and loss of tau-mediated TPI protection). That increased MG generation can also compromise the proteolysis of aberrant proteins forms [19] suggests that under these circumstances the generation of a deleterious cycle may be established in which undegraded beta-amyloid damages TPI which further enhances MG production etc. Glycolysis-induced TPI deficiency in astrocytes can explain the beneficial effects of aerobic exercise towards age-related neuronal dysfunction by providing muscle-derived lactate as an alternative energy source for brain mitochondria. It is furthermore proposed that excessive and persistent glycolysis in erythrocytes can also induce MG generation and glycated alpha-synuclein. Under such circumstances it is possible that erythrocytes may be systemic sources of both MG and glycated alpha-synuclein. Conversely, dietary-mediated glycolytic suppression would decrease the likelihood some of these outcomes.
